# Implementation of the Family Nurse Partnership programme in England: experiences of key health professionals explored through trial parallel process evaluation

**DOI:** 10.1186/s12912-019-0338-y

**Published:** 2019-04-02

**Authors:** J. Sanders, Sue Channon, Nina Gobat, Kristina Bennert, Katy Addison, Mike Robling

**Affiliations:** 1 Clinical Nursing and Midwifery, School of Healthcare Sciences, Room 1.7, Ty Dewi Sant,Heath Park, Cardiff, CF14 4XN UK; 2 Centre for Trials Research, Cardiff University, Cardiff, CF14 4YS UK; 3 Division of Population Medicine, Cardiff University, Cardiff, CF14 4YS UK; 4 Department of Psychology, Clinical Psychology, Bath, Somerset BA2 7AY UK

**Keywords:** Family nurse partnership, Teenage, Pregnancy, Early years, Home visiting, Public health nursing, Complex intervention, Process evaluation, Qualitative focus groups

## Abstract

**Background:**

The Family Nurse Partnership (FNP) programme was introduced to support young first-time mothers. A randomised trial found FNP added little short-term benefit compared to usual care. The study included a comprehensive parallel process evaluation, including focus groups, conducted to aid understanding of the introduction of the programme into a new service and social context. The aim of the focus groups was to investigate views of key health professionals towards the integration and delivery of FNP programme in England.

**Methods:**

Focus groups were conducted separately with Family Nurses, Health Visitors and Midwives at trial sites during 2011–2012. Transcripts from audio-recordings were analysed thematically.

**Results:**

A total of 122 professionals participated in one of 19 focus groups. Family Nurses were confident in the effectiveness of FNP, although they experienced practical difficulties meeting programme fidelity targets and considered that programme goals did not sufficiently reflect client or community priorities. Health Visitors and Midwives regarded FNP as well-resourced and beneficial to clients, describing their own services as undervalued and struggling. They wished to work closely with Family Nurses, but felt excluded from doing so by practical barriers and programme protection.

**Conclusion:**

FNP was described as well-resourced and delivered by highly motivated and well supported Family Nurses. FNP eligibility, content and outcomes conflicted with individual client and community priorities. These factors may have restricted the potential effectiveness of a programme developed and previously tested in a different social milieu. Building Blocks ISRCTN23019866 Registered 20/04/2009.

## Background

The US-developed Nurse-Family Partnership® (NFP) program, is an intensive, structured home visiting service aimed at improving the life chances of young parents and their children. Three US trials collectively found NFP to improve aspects of prenatal health behaviours, child care, child and adolescent functioning and maternal life course [1–9]. NFP has developed a four stage model for international replication: adaptation to local need, piloting, evaluation and wider implementation. Following this model the renamed, UK-adapted Family Nurse Partnership (FNP) programme was introduced in 10 pilot sites in England in 2007. Evaluation of 10 pilot sites established that the programme was deliverable in the National Health Service (NHS) in England, acceptable to clients, challenging but popular with the Family Nurses delivering the programme, and showed evidence of potential effectiveness [10–12]. A randomised trial, Building Blocks (ISRCTN23019866), was commissioned to test the effectiveness of FNP in England [13]. The Building Blocks trial recruited 1645 pregnant teenagers from 18 primary care sites across England [14] and found delivery of FNP yielded little short-term benefit above pre-existing care provision alone.

Policy makers have access to a global body of evidence from which to select interventions for local or national commissioning. All interventions have a finite potential to influence the outcomes of interest and it is recognised that this potential may attenuate when programmes are expanded outside of the tightly controlled research environment of programme developers [15]. Reasons proposed for reducing programme effectiveness when programmes are scaled up include: extending client eligibility to include those at less risk or with greater heterogeneity; insufficient supportive service infrastructure; and loss of programme fidelity [15]. When an intervention, previously demonstrated to have been effective, fails to influence outcomes following geographic or cultural transfer, process evaluation has an important role in providing insights into the identified gap between the expected and realised results.

The MRC guidance on the process evaluation of complex interventions [16] recognises that the intervention, implementation, mechanisms and context of intervention delivery all interact to influence outcomes. Using this model, in parallel to the Building Blocks trial [14] we conducted a process evaluation, using a convergent mixed methods design in which quantitative and qualitative data were collected concurrently, analysed separately and then considered together at the interpretation and discussion stage. The framework was prospectively developed to monitor and document programme and trial fidelity, participant engagement, recruitment and retention to the trial, contamination between trial arms and the impact of context on implementation and outcomes. A key component of this exploration of context was focus groups with health professionals which may now aid understanding of the limited programme short-term effectiveness seen in England, compared to that anticipated.

### Aims

A component of the trial parallel process evaluation aimed to investigate views of key health professionals towards the implementation, mechanisms of influence, social and service context of the Family Nurse Partnership (FNP) programme in England and contribute to interpretation of the Building Blocks trial results.

Building on the implementation evaluation of FNP in 10 pilot sites by Barnes and colleagues [10–12] the Building Blocks Process Evaluation aimed to: 1) identify contextual factors affecting implementation of FNP within the context of the RCT, 2) capture factors that may have impacted on trial outcomes, and aid our interpretation of these (i.e. factors impacting on engagement/attrition of clients and fidelity of programme delivery) and 3) document factors relevant to the wider roll-out of the FNP programme, such as workload issues, team morale, and the interface with universal services.

## Methods

### Setting

All 18 trial sites were partnerships of primary and secondary local NHS organisations and local authorities and had applied, with others, to the Department for Health to deliver FNP and additionally agreed to participate in the trial. Successful sites were selected against set eligibility criteria, and were required to deliver FNP and participate in the trial. Each of the ten contemporary English strategic Health Authorities was represented by at least one trial site.

All Family Nurses have active registration with the UK Nursing Midwifery Council as a nurse, midwife, or both, and some also hold registration as a specialist community public health nurse (formerly Health Visitors, and still commonly referred to as such).

All trial participants continued to receive usual care provided by NHS maternity services, including, according to clinical need, community and hospital based antenatal care, and community based postnatal care throughout pregnancy and until around 28 days following birth. For women in the ‘usual care arm’ of the trial, from around 10 days after birth until school entry, Health Visitors had responsibility for the provision of community based specialist public health support to families. For women in the FNP trial arm, FNP was provided in addition to midwifery care during pregnancy, but replaced Health Visiting in the period from 10 days following birth until around the time of the child’s second birthday. Thus for families in the FNP trial arm, Health Visitors required a ‘hand-over’ from Family Nurses once the FNP programme concluded around the time of the child’s second birthday. Because all trial participants received community based midwifery care, and Health Visitors continued to care for families in the trial either from 10 days post birth, or once the FNP programme concluded, Midwives and Health Visitors were identified as the two key community based health professional groups most closely aligned to Family Nurses.

Anecdotal discussions during earlier site visits had highlighted feelings that FNP was receiving funding and staffing in preference to, and resulting in strain on, usually provided services. Thus, the decision was made to run uni-professional focus groups with Family Nurses, Midwives and Health Visitors to allow for a full exploration of the service context from multiple perspectives e.g. the relationship between the different teams and the experience of FNP. Focus groups were organised through professional managers and could include any member of the team. Wherever possible the focus groups were arranged to coincide with the venue and timing of professional team meetings but in a few cases meetings were organised as stand-alone events. Maximum variation sampling was used to determine eight focus group sites from the 18 sites, with sampling criteria (set out in Tables 1 and 2) chosen to maximise diversity of the professionals’ experiences in terms of timing of site opening, context of service and trial recruitment. Therefore, sampling criteria reflected established or newer FNP teams, urban / rural diversity and differing levels of participant recruitment to the trial. Four sites were selected that opened in 2008 when FNP was first established in England (FNP Wave 1), with the other four sites having opened in 2009 as part of FNP expansion that accompanied the trial (FNP Wave 2). The sites identified using the sampling criteria were contacted. All sites and all professional groups contacted agreed to participate in the focus groups. The participants were invited to attend by the professional leads. For Midwives and Health Visitors eligibility for participation in a focus group was experience of caring for clients who had received FNP. All Family Nurses at focus group sites were eligible for participation in a focus group, including FNP supervisors. On one occasion the FNP team administrator was also present. No potential participants refused to participate, but passive non-engagement by individual participants, e.g. not attending the group on the day, was not captured.

**Table 1:** Building Blocks Focus Group sampling criteria per site

Site ID	Site sampling criteria	Professional groups	Focus group time frame
G	Wave 1, smaller city/urban character, low trial recruitment	FN, HV, MW	Round 1: 15 March to 7 June 2011 (NB low-medium-high recruitment measured in total number of women recruited)
R	Wave 2, rural, medium trial recruitment	FN, HV(× 2), MW	
N	Wave 1, smaller city/rural character, low trial recruitment	FN, HV, MW	
Q	Wave 2, large city, high trial recruitment	FN, HV, MW	
H	Wave 1, urban, late* trial recruiting site	FN	Round 2: 26 April to 25 June 2012 (all sites expected to have medium to high number of handovers at time of FG meeting)
J	Wave 1, London, early stop* trial recruiting site	FN, HV	
C	Wave 2, urban, late trial recruiting site	FN	
I	Wave 2, rural, medium length* trial recruitment	FN, HV	

^*^early/medium/late refers to length of trial recruitment period which varied across sites: early: recruitment to trial finished by end March 2010, medium: finished by end May 2010, late: finished by end June 2010

**Table 2:** Trial sites participating in focus groups

Site ID	Wave	Location	Trial participants at site	Focus groups			Time in relation to study activity	
				Family Nurses	Midwives	Health Visitors	Following completion of recruitment and births of babies	Around FNP completion (babies aged 2)
G	1	City	43	✓	✓	✓	✓	
R	2	Rural	99	✓	✓	✓ × 2	✓	
N	1	City	49	✓	✓	✓	✓	
Q	2	City	142	✓	✓	✓	✓	
H	1	City	143	✓				✓
J	1	City	47	✓		✓		✓
C	2	City	115	✓				✓
I	2	Rural	68	✓		✓		✓

### Data collection

Focus groups were held at two time periods of the trial, being conducted with the three professional groups towards the end of the two-year recruitment period and with Health Visitors and Family Nurses once they had experience of clients who had completed the programme. Topic guides for all groups included broad aspects of programme implementation, whilst leaving scope for probing and the raising of local or individual issues (Table 3). Specific issues covered in the Family Nurse discussions included motives for joining FNP, training, workloads, fidelity targets and saying goodbye to clients. Health Visitors were asked their initial views towards FNP, how they have found it varied from Health Visiting, how FNP impacted on the caseloads of Health Visitors and the quality of handover from FNP following clients’ completion of the programme. Midwives were asked about their views towards FNP, how it has impacted on their workloads and professional relationships with FNP staff. Prompts and follow-up questions explored perspectives on FNP in relation to each professional group’s understanding of their roles, values and working practices within the context of current and previous service changes and developments.

**Table 3:** Summary of key questions explored in the focus groups, and repeated across time points

Q1. How has FNP been implemented at your site? What has worked well and what has not? (key issues: signing up women, setting up issues) Q3. What has changed as a result of FNP? (e.g., with the women, service provision, ways of working across the site, professional relationships across services) Q4. What advice would you give to others considering implementing FNP?	

All focus groups were conducted by experienced qualitative researchers [MJ-B, KB]. Focus group participants were informed that the focus group conveners were members of the Building Blocks research team. Focus groups were digitally audio-recorded and transcribed verbatim and when required researches made additional field notes. Names, places and other potential identifiers were replaced with generic descriptors at the transcription stage and ID numbers were used to indicate sites.

### Ethical considerations

The trial was approved by a NHS Research Ethics Committee for Wales, reference number (09/MRE09/08). We contacted professional leads who invited their team members and circulated information sheets and consent forms before the meetings. At the start of the meeting participants re-read the information sheet, and provided written consent to participate including for audio-recording and use of anonymised data including direct quotes.

### Data analysis

Transcripts from audio-recordings were coded and analysed thematically by three experienced qualitative researchers (KB, NG, MJ-B) to produce a detailed coding framework, based on the a priori aims of the study and new themes which emerged from the data. For the Family Nurse focus group data, all transcripts were scrutinised independently by the two experienced qualitative researchers who facilitated the meetings (KB, MJ-B). At this initial stage, three broad content areas (key questions, professional perspectives and evaluation of processes), as well as initial lists of thematic codes to fit those content areas were identified. Coding of transcripts was supported by NVivo 8 and a detailed coding framework building on those initial observations was devised by one researcher (MJ-B). During the coding process, discussions about the expanding coding framework as well as the assignment of codes were held at regular intervals. These were informed by impressions (captured in field notes) taken at the time of data collection by both researchers. For pragmatic reasons, the Health Visitor and Midwife focus group data were analysed by a researcher not involved in data collection. (NG). Data were analysed thematically, with themes identified as patterns in the data in response to the research question. The coding frame was finalised after a second qualitative researcher (KB), double coded 25% of the data. Where 95% agreement was reached between the coders, no action was taken. In other cases, the coders reviewed areas of discrepancy and resolved these. There were no coding discrepancies that could not be resolved. Coding comparisons were then rerun to ensure that 95% agreement was reached across all higher-level codes.

## Results

A total of 19 focus groups were conducted across eight of the 18 trial sites: Midwives (*n* = 4), Health Visitors (*n* = 7) and Family Nurses (*n* = 8), and represented study sites in rural areas as well as small and large cities. Attendance ranged from 2 to 12 participants, ranged in duration from 60 to 75 min and a total of 122 professionals contributed. Participants generally represented highly experienced staff with an average duration since initial registration with the UK Nursing, Midwifery Council (NMC) of 21 years for Midwives, 17 years for Health Visitors and 20 years for Family Nurses. The Family Nurses working in the four FNP sites that opened in 2007 (FNP wave 1) all had two years FNP experience prior to the recruitment of women into the trial, whereas the Family Nurses working at the four wave 2 sites were new to the role, and enrolled up to five non-trial participants on the programme, prior to commencing the programme with trial participants. Four FNP supervisors (who also held a client caseload) and three FNP administrators participated in the Family Nurse focus groups, and a smoking cessation advisor and two student Midwives participated in the Midwifery discussion groups. One Health Visitor was interviewed separately. Meetings lasted between 60 and 75 min, and were facilitated by usually two, but on occasion just one, experienced qualitative researchers.

The discussions with each of the three professional groups followed similar themes reflecting the broad subjects included in the topic guides: a) how the FNP programme compared to usual Midwifery and Health Visiting; b) challenges of delivering services; c) FNP integration alongside existing services; and among the Family Nurses d) programme fidelity targets and measured programme and trial outcomes.

Higher level themes were assessed for the FNP nurses, the health visitors and midwives separately before being integrated and reported. For Family Nurses core themes centred around delivery of the programme, including what affected client enrolment and then their subsequent engagement and finally observation of the concurrent trial. Delivery considerations for Family Nurses encompassed coping with workload, especially in the context of clients with complex needs and its interaction with programme fidelity requirements. Further delivery considerations included co-located services, variations in skill and training mix within their team, achieving continuity of care and fidelity to session content, the impact of local cultural factors upon fidelity. Client enrolment and engagement considerations involved local awareness of FNP, issues related to the wave of programme rollout (and how that interacted with for example trial rollout) and specific factors that may have influenced individual client engagement.

For the Health Visitor and midwifery professionals working alongside FNP teams, the thematic focus was more upon their external experience of FNP – their experiences of FNP professionals and teams, their experiences how FNP interfaced with their own and other services and also experience of the trial. Amongst experiences reported about FNP, focus group participants addressed their perceptions of the programme content, the nurses’ role, its implementation and impact and implications for usual care. Interface issues include matters of client referral, inter-professional working and client handover at the end of the FNP period of support.

In integrating these themes, we have identified four focal points. First, were experiences within services and the contrast between FNP and usual care. Encompassed within this was matters related to resourcing (including differences across professional services), variations in intensity and time available for client support and matters of professional competency. Second, the interaction between FNP and usual care was explored. This covered practical and logistical matters such as record sharing, co-location and resource sharing. Third, and a focus particularly for FNP staff was matters related to FNP programme adherence. This addressed matters such as the impact upon practice of applying programme eligibility criteria (including how this may also the impact upon fidelity attainment). This may also then lead to some questioning of the suitability of such criteria. Observations about FNP adherence were more directly reportable by FNP teams for whom this topic was most directly relevant. The fourth focal point addressed the concurrent trial and observations about facets such as relative value of different perceived outcomes. Each focal point so described in more detail below.

### Working as a family nurse work compared to usual care

Midwife and Health Visitor groups expressed mixed feelings about the new professional role of the Family Nurse. Many expressed positive views about FNP with lower caseloads and generous investment in equipment and training.

HV 1: I- I was jeal- of FNs, really, that um, that they could spend

HV 3: Yeah.

HV 1: all that time with clients, and, and thinking that really this is what we should be doing.

(HV 3 and 4 agree))

HV 2: I think I felt quite excited, at the thought of it coming, [Site I]

A Midwife described their service being like a ‘poor relation’ that had been ‘abandoned’. Consequently, FNP was experienced as a threat, challenging the perceived competence of Midwifery and Health Visiting.

MW: It was like they were something better, and wonderful, and were going to offer a service, which was a bit of a kick in the teeth when we’ve all worked so hard. [Site R]

Family Nurses, were aware their small caseloads were the subject of the envy of other colleagues but felt this reflected a lack of appreciation of the intensity and demands of their work.

FN : Health Visitors will say ‘I’ve got 800 families you’ve got 25 don’t talk to me about work’

FN : We are working very long hours and a lot of our time that- we do computer work and what have you, and data is all done in our own time, a lot of it is. [site J]

The 3 weeks residential based training provided to Family Nurses was in stark contrast to the restrictions being placed on Health Visitors attending training during the same time periods.

FN: I think the other tricky bit was there a moratorium on training when we came in to FNP for Health Visitors on the ground they were only allowed to do mandatory training. There was no money to do anything else over and above, so I think they were some sort of envy about the amount of training we were having and the weeks away and stuff like that. [Site G]

The smaller caseloads held by Family Nurses enabled FNP clients to be offered the high intensity of support intended:

FN 4: as a Health Visitor for example you might see a client three times we would see somebody sixty-three times so the depth and amount of information we have to disseminate is huge. [Site Q]

Despite describing their workloads as demanding, Family Nurses were positive about the programme, confident in its effectiveness and considered the ‘strengths based approach’ of the programme represented a fundamentally different approach to clients compared to that of Midwives and Health Visitors.

FN 4: you’re trying to come in with a completely different approach that you know works. Uhm, and I think sometimes other professionals think you’re on another planet in terms of the way you approach things.

FN 2: Because we talk about strengths, don’t we? [Site H]

### Integration with existing services

Whilst some sites reported very positive working relationships, in others poor communication between FNP and usual care services was evident with, for example, FNP clients choosing to attend a regular ‘well baby clinics’ but the attending Health Visitors not having access to records relating to the family.

HV 7: So you would get someone coming to clinic, and you would have no background information about them at all, except maybe what’s written in the Red Book [child health record held by the mother] but that would be limited. [Site J]

Poor communication was particularly evident in areas where FNP teams had been based in separate locations from existing services, and more effective where staff were co-located.

HV 9: My experience is that they work in the office next door to us so we have a lot of dealings with them um we share the same sort of catering facilities we chat over lunch and they are a good group of girls and they work very, very hard. [Site Q]

MW 1: In an ideal world we would be having offices not far from each other, maybe sharing a kitchen and popping in and saying 'oh hello, how are you, have you seen so and so'. [Site N]

Health Visitors expressed a desire to be better informed about FNP and to have access to FNP materials. Family Nurses expressed that the integrity of the programme needed to be protected and that ‘leakage’ of programme materials and methods into usual care might breach the conditions of the FNP licence and jeopardise the trial’s ability to demonstrate FNP effectiveness.

HV 1: I’d like to see more integration and more working with us and using them, the fantastic ways in which the service work with us and becoming part of our service, a real part of the service, you know, (unclear) really, families that benefit can actually be put on and FNP can work with them not instead of us but with us. [Site Q]

One recently qualified Health Visitor had requested to join the team of Family Nurses for a day during her training but this had been declined on the grounds of protecting programme materials. A Family Nurse described how they were instructed in training not to share FNP materials:

HV 4: I only qualified in September, I know very little about the FNP, I did try and go out with them for a day in the training, but, they weren’t allowed to do that because of it being a trial. [Site R]

FN 1: In the pregnancy training we were kind of, like, told, oh, you can’t share this with anybody, because it’s a licensed programme, so I felt when I went back, like I had this big secret. [Site C]

Health Visitors and Family Nurses described how services had actively engaged in the separation of trial participants in the treatment as usual and FNP arms. Some Health Visitors mistakenly thought that to militate against them enhancing their usual care, they were not to know who on their caseload was in the control group of the trial:

FN 4: I think we were discouraged really from bringing them [RCT clients and their friends] together. And right at the very beginning we were discouraged, really, from letting them share what we were doing with friends who weren’t doing it, weren’t we? [Site C]

HV 2: I thought that we weren’t supposed to know. Otherwise, if we knew that they were on the trial, um as, you know, what do you call it, as a placebo or whatever, then we may be treating them differently, us giving them a different service then perhaps our other caseload. [Site N]

One Health Visitor participant, who had previously worked as a Family Nurse, considered that protecting FNP materials was unnecessary. She thought much of the knowledge base of FNP already existed in Health Visiting, but with Heath Visitors having neither the time nor the resources to provide a similar level of care to that being offered within FNP.

### Adherence to the FNP programme

Family Nurses questioned the fidelity targets of the programme around enrolment criteria and delivery. The trial eligibility criteria reflected those of FNP in England, recruiting teenagers expecting their first baby and living in a socially deprived area which offered the programme. Although most FNP clients were regarded as in need, some were felt not to have required such intensive support, whilst others, either due to a chaotic life style or limited previous education, were considered to require more input than the programme allocated.

FN 4: some girls that you knew would benefit from FNP wouldn’t get it.

FN 2: some girls that got it perhaps didn’t need it or didn’t make the best use of

FN 4: and I think that one of the things that I have noticed is that some of the girls that have been recruited to the trial is ones that are more .. well-resourced [Site Q]

A midwife supported the idea that some FNP clients may not require the intensity of support offered.

MW : There’s a lot of people [who think] having babies at seventeen, eighteen is normal and they don’t see what the big deal is, and they’re not probably people who in my opinion would benefit from such intensity because they don’t see that as an issue. … A lot of the young girls, their parents have had babies at sixteen, seventeen they’re having babies again, that's the norm, that's what’s expected of them and they have got family support. [Site R]

In one site Family Nurses indicated that programme delivery was more challenging because Midwives were referring to FNP ‘high need’ clients with ‘chaotic’ lifestyles.

Family Nurses reported difficulties in maintaining aspects of programme fidelity, and were aware their self-reported adherence was subject to scrutiny locally and by the FNP national unit. A programme requirement is that the client’s participation is voluntary. Although this was always the case at enrolment, the requirement was breached on occasions due to social services mandating continued FNP engagement.

FN: They [FNP clients] have multiple appointments and it seems to be a case of fitting them all in. They see social care as their main priority because they have got to fulfil all those appointments, you see, and because our service is voluntary it’s like an add-on, so something might have to give and it might be Family Nurse Partnership. But, on the other hand, social services will say, ‘No you have got to have Family Nurse Partnership, so then it becomes not voluntary, it becomes something that they have to do. [Site G]

Family Nurses across all sites reported greater difficulties with arranging the required number of visits towards the end of the infancy period, although this was viewed as positive reflecting clients’ engagement with work or education.

FN: I’ve got a girl in university I’m very proud of, and she’s cancelled me today because 'I’ve got an assignment to do', and it’s like I really struggle to get in to her. But it’s not because there’s stuff going on, it’s just because she’s got her head down and she’s getting her uni work done and I know (FN name) has the same problem with a girl that’s in college or in school. [site H]

An important component of FNP is the therapeutic relationship developed between the nurse and client. For this reason, continuity of the Family Nurse is a programme fidelity target. All focus group sites described experience of Family Nurses needing periods of long-term sick or maternity leave resulting in clients changing their Family Nurse, in some cases more than once. Midwives providing care to FNP clients recognised disappointment when continuity of their Family Nurse could not be maintained, or the programme was disrupted.

MW4: Because one girl I have just delivered, her Family Nurse has gone off on long term sick and I know that she found that quite, um, well, it’s a disappointment … I mean, it can’t be helped if it is long term sick, but I wondered if there would be a team rather than - as after you have built up that relationship - I know what a young girl would feel like. They would be disappointed, wouldn’t they? [Site G]

Family Nurses from several teams described difficulty keeping to prescribed programme content or recommended domain proportions when other client concerns dominated:

FN 1: From the studies in the States, I don’t think those nurses have the same issues with the environment part of our visits because when you get your reports back you’re only supposed to spend a specific amount of time discussing issues around the environment, so when we get our report ours are always in the red to say we’re spending too much time in the environment, but those are the issues that the girls are presenting with, you know, housing

FN 4: It’s particular for this area.

FN 1: yeah housing, homelessness, … [site H]

### Programme and trial outcomes

Family Nurses expressed concern that the trial was measuring rates of smoking, breastfeeding and subsequent pregnancies. All these programme objectives were regarded as challenging to influence due to the prevailing cultural and intergenerational norms in FNP areas and were not regarded as the highest priorities for clients. Family Nurses also expressed that the trial was not assessing other outcomes that they considered to be improved by the programme.

FN1: We have got a lot of intergenerational smoking so it's a challenge to work on smoking reduction when everybody in the family smokes. [site Q]

FN 1: because some of the health outcomes, we’re looking, you know, they always tend to look at breastfeeding and smoking. I’m not saying they’re not important, but the kind of level of work we’re doing, it should be like looking at is this mum keeping her baby,

Group: mmm, yeah

FN 1: it’s really deep, complex things like

FN 6: is the dad staying out of prison. [site H]

## Discussion

FNP was introduced into England with a high expectation of short-term effectiveness. As a component of the study’s process evaluation we conducted focus groups with key health professionals and these can now aid understanding of why the programme yielded little additional short-term benefit over existing services in England.

A failure to provide sufficient service infrastructure ‘on the ground’ can result in interventions not being delivered as intended [15] with programme dilution of dose and effectiveness. However, FNP implementation benefited from a structure of local teams including FNP supervisor and administrative support, sufficiently resourced to offer the intended intensive programme to vulnerable young families. Unusually for England, a senior central team initially based at the Department of Health oversaw the programme’s introduction and expansion, ensuring consistency of training and resourcing of teams across NHS providers. This centralised administration, although bringing benefits of consistency of training and the development of a cohesive national professional identity for the Family Nurse teams, contributed to the service as being seen as elitist. Instructed by the central team to protect the content of the licenced programme, Family Nurses felt unable to share programme materials with colleagues outside of FNP. This is unusual behaviour in England where locally developed materials are frequently shared across the NHS upon request. This issue led to some resentment from the Health Visitors who as well as being unable to access levels of training and support provided to Family Nurses, were not permitted to have sight of FNP materials which they considered could have enhanced their own practice and understanding of the FNP programme.

It is a prerequisite of sites wishing to implement FNP to demonstrate capability to operate and sustain the programme to a high quality [17] and we found no suggestion that a lack of infrastructure had adversely affected the implementation of FNP.

An intervention may fail to show benefit if it is insufficiently different from the control conditions. All professional groups agreed that FNP represented a substantial increase in input for clients compared to usually provided services. This opinion was supported by trial evaluation which found although women in both trial arms received a mean 10.7 community midwifery contacts during pregnancy, FNP clients received an average of 41.5 home based visits with an average duration of 79.14 min during pregnancy and until the child’s second birthday, compared to 5.1 home visits from Health Visitors received by control group participants over the same period.

Family Nurses described the challenge of providing a client led intervention while adhering to programme fidelity requirements of the licenced programme, finding at times that the programme was not responsive to the needs of clients. The tension between adherence to manualised programmes whilst addressing individual client need is recognised [15, 18] and requiring strict adherence to manualised programme content has been challenged on the grounds of limiting client or shared agenda setting. Although FNP recognises the need for programme flexibility, nurses are required to allocate prescribed proportions of visit time to each of the programme’s five domains. Family Nurses found the proportion of time allocated to ‘environmental health’ to be inadequate, and described difficulty in delivering programme materials when clients had other priorities.

The programme fidelity requirements of nurse continuity and the voluntary nature of programme engagement were challenging to deliver in practice. The long term therapeutic relationship between Family Nurses and clients are regarded as fundamental to the programme, and necessary to address ‘difficult’ issues including domestic abuse [19]. For 30% of FNP clients in the trial, life events such as maternity leave or sickness led to visits being undertaken by more than one, and for a small number, up to five nurses [20]. The voluntary nature of the programme was for some compromised by the mandating of FNP clients to continue with the programme as a requirement of child safeguarding proceedings. For such clients, this lack of nurse continuity or the mandating of the programme may have had a negative effect on their receptiveness to the programme.

During programme initiation in England it was recommended that FNP use young maternal age as a pragmatic indicator of risk and eligibility [21] and this was subsequently incorporated in trial eligibility. The decision to use age alone was considered by some focus group participants to have resulted in some less disadvantaged young mothers entering the programme, with this occurring even when FNP was only offered to women living in areas of recognised social deprivation. Evaluation in the Netherlands, where the programme is named VoorZorg, found positive programme effects, including reduced domestic violence and rapid subsequent births [17, 22–24]. Amongst Building Blocks trial participants these important programme outcomes were unaffected by FNP.

In the US, NFP was found to be of greatest benefit when provided to women with low psychological resources [25] and the Netherlands used additional screening to identify women most at risk, but with a broader age range eligibility of up to 26 years. Despite all being under 20 years of age at recruitment, it is possible that FNP trial participants were at less risk and more heterogeneous than those in the US or Netherlands trials [26]. Additional screening could be used in England to identify women more likely to benefit from FNP, but a priori sub-group analysis of trial data did not suggest this approach would increase effectiveness [20].

Focus group participants across the three professional groups identified that teenage motherhood was the accepted norm in many areas. Despite recent falling rates, the UK still ranks fifth highest amongst 28 European countries with a rate of live births to teenagers of 13.7 /1000 in 2016 [27].

The system of usual care into which FNP was added was itself complex [28] with women having access to multiple other health and social care services. This level of background or usual care was in stark contrast to ‘usual care’ experienced by participants of the US trial of NFP, where participants receiving ‘usual care’ received no community based midwifery or public health nursing services and few services provided by statutory or voluntary organisations.

Overlaying of the US modelled NFP onto existing services in England led to inefficiencies and FNP clients needing to engage with more services than necessary. Family Nurses engage with clients generally considered ‘hard to reach’ by supportive services so the exclusion of some aspects of clinical care from FNP delivery in England including early practical breastfeeding support and contraception provision were missed opportunities to maximise the programme’s effectiveness.

Family Nurses considered some of the programme goals, including those targeting smoking and subsequent pregnancies, were not reflective of the hierarchy of needs of clients, and failed to recognise the complexity of modifying intergenerational behavioural norms. To enable engagement with education or employment, and to reduce parental strain, a programme aim for clients is to achieve longer birth intervals and fewer total numbers of children. Family Nurses spoke of this programme aim as conflicting with the social norms of clients’ communities in which young motherhood and closely spaced children were expected and desired. The desire to have further children at a young age, and Government provided financial support for families, may mean FNP clients in England are less motivated to extend inter-birth intervals than participants in the US trials [29, 30]. Similarly, Family Nurses considered the programme goal of reducing smoking challenging due to the normality of smoking, and its intergenerational nature, in communities served by FNP. High rates of smoking in late pregnancy and subsequent pregnancies were found in both FNP and control arms with no evidence of programme effect.

It is the very challenging nature of influencing life style choices such as smoking and repeat pregnancies that forms the justification for the intensity of the FNP programme. In the UK context, if other insurmountable issues, such as housing insecurity, act as inhibitors to FNP programme effectiveness, investment in social housing and other infrastructure may be a necessary prerequisite, or a more effective alternative, to FNP.

In the interpretation of a lack of short-term FNP effectiveness, it is important to consider if the intervention group received sufficiently different care from controls. Some participants allocated to usual care were exposed to FNP-trained staff, or clients of FNP. As the programme’s effectiveness is founded in the deep therapeutic relationship [31] between client and Family Nurse, regular visits and prolonged programme exposure, the apparent minimal levels of exposures to FNP materials experienced by trial participants allocated to usual care, are considered unlikely to have been effective in modifying outcomes, or responsible for the negative trial results.

The focus group discussions touched upon a wide range of issues relating to the provision of the FNP programme in England. To some extent these discussions may have been curtailed by the a priori setting of research questions, with the reporting reflecting subsequent prominent themes. The 122 health professionals that participated in study focus groups worked in eight of the 18 trial sites and included representatives of all members of the team. This enabled multiple perspectives to be included and there was no evidence that having the supervisor of the team present restricted discussion, but this could have been possible. Although analysis found consistency of themes across participating sites, other issues may have been pertinent in other trial sites.

The experience in England demonstrates the importance of the NFPs recommended stages for international replication. In England adaption and piloting of the programme were conducted, but during the trial, FNP was expanded from 18 to over 110 sites. Subsequently the trial demonstrated limited programme effectiveness. Although longer term FNP programme effectiveness may yet be demonstrated, programme expansion was not justified during the period of the trial due to a lack of locally relevant evidence, and this may over time, be viewed as an ineffective use of public monies.

The process evaluation conducted in parallel to the Building Block trial had several strengths. It was designed and facilitated by an independent team of experienced qualitative researchers. Maximum variation sampling ensured the voices of practitioners in a range of practice settings were heard. FNP introduction represented a major investment in the provision of care to young families in the study sites. Hearing the voices of health visitors and midwives whose services needed to work alongside FNP, but without similar investment, was important. The three professional groups independently described FNP as being far more intensive, from that which was previously provided to young families.

## Conclusion

When public health interventions fail to deliver expected improvements following project expansion, process evaluation can aid understanding of the intervention and interaction with usual care. FNP at trial sites was described as supported by a well-resourced infrastructure, and delivered by motivated Family Nurses convinced of the programme’s effectiveness. Key professional groups identified factors that may have limited the ability of FNP in England to demonstrate the expected short-term benefits over usually provided services. Entrenched cultural norms associated with low motivation to change, combined with recruitment of some lower risk clients were identified by the focus groups as issues they encountered during the Building Blocks trial. These issues may have contributed to the programme’s inability to influence key programme outcomes including smoking during pregnancy, birthweight, inter-pregnancy interval and child emergency attendances or admissions to hospital within the child’s first two years [14]. For FNP programme continuation to be justified in England, the ongoing evaluation of longer term programme goals such as reducing child neglect, and improved child development will need to demonstrate effectiveness [32], or the FNP Next Steps and ADAPT programme amendments (https://fnp.nhs.uk/fnp-next-steps/adapt/) made subsequent to the trial results, will need to demonstrate influence on existing short-term programme goals including smoking during pregnancy and subsequent pregnancies.

## Abbreviations

FN: Family Nurse
FNP: Family Nurse Partnership
HV: Health Visitor
MRC: Medical Research Council
MW: Midwife
NFP: Nurse Family Partnership
NHS: National Health Service
RCT: Randomised Controlled Trial
UK: United Kingdom
US: United States of America
